# A Rare Triplet Found in a Patient With Drug-Resistant Hypertension: Adnexal-Adrenal Sex Cord-Stromal Tumors and Castleman’s Disease

**DOI:** 10.7759/cureus.28220

**Published:** 2022-08-20

**Authors:** Gurcan Erbay, Umur Anıl Pehlivan, Elif Karadeli

**Affiliations:** 1 Department of Radiology, Başkent University Faculty of Medicine, Adana, TUR; 2 Department of Radiology, Van Baskale State Hospital, Van, TUR

**Keywords:** mri, genitourinary imaging, castleman’s disease, ovarian sex cord-stromal tumor, adrenal sex cord-stromal tumor

## Abstract

Adrenal and ovarian sex cord-stromal tumors which originate from the ovarian stroma and adrenal cortex, have an extremely low incidence even alone. Castleman's disease is also one of the rare causes of non-neoplastic lymphadenopathy. The aim of this case report was to contribute to the literature by identifying the coexistence of these three pathologies, which are encountered with a low incidence even alone.

A 46-year-old female patient had experienced drug-resistant hypertension. In the laboratory test, electrolyte imbalance was detected. Imaging modalities revealed left adrenal and adnexal mass lesions as well as a right paraspinal intramuscular nodular lesion. When hypertension and electrolyte imbalance were evaluated together with their etiology, it was thought that the left adrenal lesion, which also has a chemical shift, may be compatible with functional adenoma. Left adrenalectomy was planned. The ovarian lesion and right paraspinal intramuscular lesion were also excised during the same session with the suspicion that the ovarian lesion may be hormone-active neoplasia and the intramuscular lesion may be a metastasis. The diagnosis of adnexal-adrenal sex cord-stromal tumors and unicentric Castleman’s disease were made histopathologically.

Radiologically, it should be kept in mind that the radiological manifestations of adrenal sex cord-stromal tumors can be confused with adrenal adenomas. Even when these extremely rare tumors are encountered, avoiding the "satisfaction of search" plays a crucial role in the identification of additional pathologies that can explain the etiology.

## Introduction

Sex cord-stromal tumors of the ovary, which originate from the ovarian stromal cells and primitive sex cords, are rare neoplasms that account for 8% of all ovarian neoplasms [1]. Sex cord-stromal tumors of the ovary occur in any age group and make up the vast majority of hormone-active ovarian tumors [2].

Sex cord-stromal tumor of the adrenal is a rather rare tumor of the adrenal cortex, of which only six cases have been identified so far according to the last edition of the World Health Organization (WHO) classification of tumors of endocrine organs [3]. These neoplasms are manifested as virilizing findings, abnormal uterine bleeding, persistent hypertension, and intraabdominal mass.

Castleman's disease, one of the causes of non-neoplastic lymphadenopathy, may occur at any site of the body and may mimic infectious-inflammatory and malign processes [4]. Castleman's disease is usually presented with mediastinal, intraabdominal, and head-neck lymphadenopathy. But rarely, involvement of subcutaneous or muscular structures can also be observed [5].

The aim of this case report was to contribute to the literature by identifying the coexistence of these pathologies independent of each other, which are caused by hypertension and are encountered with a low incidence even alone.

## Case presentation

A 46-year-old female patient, who had been admitted to another medical center with drug-resistant hypertension and electrolyte abnormalities, had been referred to our center with a pre-diagnosis of hyperaldosteronism. She was a homemaker and had no known systemic diseases except hypertension. She had a history of smoking half a pack a day for 25 years. Diagnosis of hypertension was achieved 12-13 years ago. But recently, regulation of hypertension could not be achieved despite the use of dual antihypertensive (nebivolol 5 mg; enalapril 10 mg). Her Eastern Cooperative Oncology Group performance scale was 1. On physical examination, there were no abnormalities except hypertension (200/100 mmHg). Laboratory tests showed hypokalemia (2.2 mmol/L) and a mildly elevated fasting blood glucose level (110 mg/dL). Outcomes of dexamethasone suppression test and urine hormone levels were normal. Potassium citrate effervescent and spironolactone were started immediately. After this treatment, the regulation of the blood pressure was achieved.

**Figure 1:**
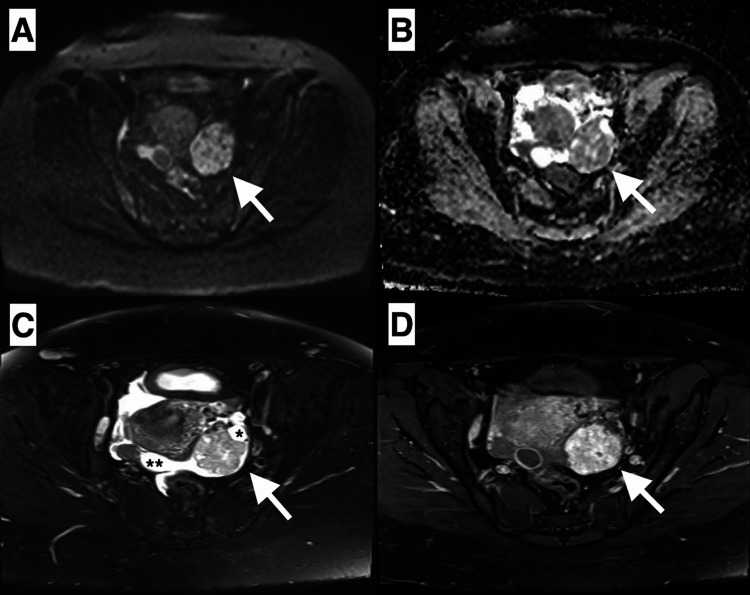
Diffusion restriction in solid component of the left adnexal mass revealed on the diffusion-weighted image and apparent diffusion coefficient mapping (A,B). A heterogeneous mass (arrow) with solid and cystic (*) components, pelvic free fluid (**) were revealed on fat-sat T2-weighted images (C). After gadolinium administration, fat-sat T1-weighted image revealed heterogeneous intense enhancement in the solid component of the left adnexal mass (D).

**Figure 2:**
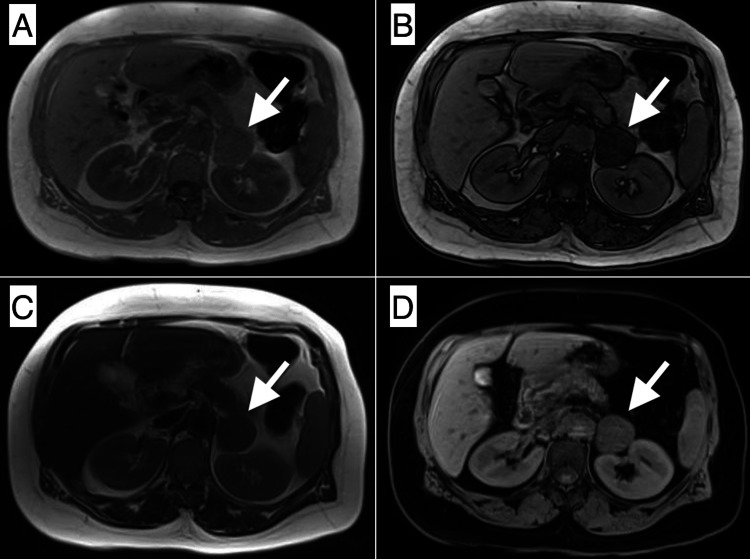
A left adrenal mass (arrow) that had the chemical shift on the in-phase (A) and out-of-phase (B) sequences and was slightly hypointense on the T2-weighted image (C) and post-contrast late-phase T1-weighted image (D) revealed.

**Figure 3:**
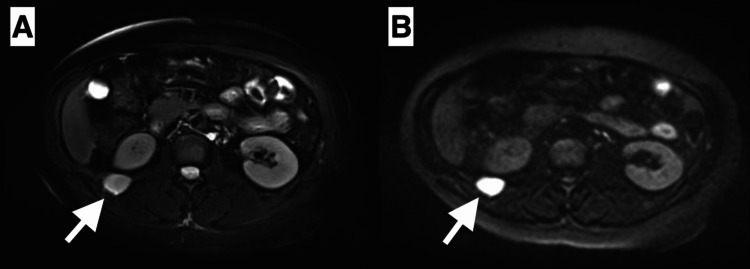
A right paraspinal intramuscular nodule (arrow) that has hyperintensity on fat-sat T2-weighted image (A), and has diffusion restriction on the diffusion-weighted image (B) was revealed.

The renal artery Doppler ultrasound was normal. Oriented to the pre-diagnosis of hyperaldosteronism, abdominal magnetic resonance imaging was performed. A heterogeneous left adnexal mass that had solid and cystic areas, radial scar-like centripetal linear fibrotic areas on T2 weighted images, diffusion restricted and intensely enhanced in the solid zone, and free pelvic fluid were observed in the lower abdominal scanning (Figure 1). A homogeneous left adrenal mass that had a chemical shift, wash-out, and restriction of diffusion, and a right paraspinal intramuscular nodule that was homogeneously enhanced and was diffusion restricted were observed in the upper abdominal scanning (Figures 2-3). In the course of the search for hypertension etiology, we had considered that the left adrenal lesion could be a functional adenoma and, that the left ovarian lesion could be a sex cord-stromal tumor. We could not make a differential diagnosis for the right paraspinal intramuscular lesion, but we also could not rule out possible metastases.

On the tumor board, an operation decision was made for the left adrenal lesion. Regarding the left adnexal mass and right paraspinal nodule, a simultaneous operation decision was made also due to atypical radiological morphology.

The pathological evaluation of the left adrenal lesion was reported compatible with a granulosa cell type of sex cord-stromal tumor. Similarly, pathological evaluation of left adnexal lesion was also compatible with a steroid cell type of sex cord-stromal tumor. The right paraspinal intramuscular nodule also was excised, this lesion's pathological evaluation was reported as the hyaline vascular type of Castleman's disease.

These three extremely rare pathologies were treated successfully in only one surgical procedure. There was non-necessity for additional treatment due to the low stages of tumors. The regulation of hypertension was achieved after the surgery.

## Discussion

Sex cord-stromal tumors of the ovary occur in any age group and make up the vast majority of hormone-active ovarian tumors that have estrogenic or virilization effects [2]. The vast common subtypes of this tumor group are granulosa cell tumors, fibrothecomas, sclerosing stromal tumors, and Sertoli-Leydig cell tumors [1,2]. As in this case, there are also steroid cell type sex cord-stromal tumors that are rare. Steroid cell sex cord-stromal tumors generally have a virilizing effect, but rarely have also Cushing-like effects. Owing to some clinical and imaging features, this rare subtype can be distinguishable from the other ovarian neoplasms [1]. Radiologically, they have hyperintense areas in T1-weighted images owing to lipid components and are intensely enhanced after contrast administration. They are generally unilateral masses that are smaller than three centimeters at the time of diagnosis. As opposed to the literature, this case contained cystic-solid components without any lipid components. So, we could not observe hyperintense areas in pre-contrast T1-weighted images. However, solid components were intensely enhanced on post-contrast T1-weighted images. On T2-weighted images, there were radial scar-like centripetal linear fibrotic areas in solid components. Probably owing to the hypercellular structure, diffusion restriction was observed on diffusion-weighted images (DWI).

Of the six sex cord-stromal tumors of the adrenal gland identified so far according to the last edition of the WHO classification of tumors of endocrine organs, three were granulosa cell tumors and the other three were Leydig cell tumors. All cases were observed solitary and unilateral in postmenopausal women. Leydig cell tumors are presented with virilizing findings, while granulosa cell tumors are presented with abnormal uterine bleeding, abdominal mass, and persistent hypertension [3]. It appeared as a unilateral adrenal mass that caused persistent hypertension in this case report. This case was the second case in the literature of granulosa cell adrenal sex cord-stromal tumor characterized by persistent hypertension [6]. Magnetic resonance imaging findings of previously documented six adrenal sex cord-stromal tumors have not been identified [6-11]. In this respect, the radiological features of this case provide a new perspective to the literature. However, radiological features of this adrenal lesion such as diffusion restriction on DWI, chemical shift on the dual-echo sequence, and wash-out on dynamic T1-weighted images, mimicked functional adrenal adenoma. Radiologists should more carefully interpret patients with suspected sex cord-stromal tumors due to clinical and laboratory results that have similar morphology to adrenal adenoma in imaging modalities.

Castleman's disease can present with two morphological subtypes including unicentric focal lesions which can be controlled by only surgery or multicentric involvements which need systemic aggressive treatments [4]. Castleman's disease is usually characterized by the primary involvement of lymph nodes in the mediastinal, intraabdominal, and head-neck areas. But rarely, as in this case, involvement of subcutaneous or muscular structures can also be observed [5]. Castleman's disease can mimic malignant processes due to its intense contrast in imaging modalities. On T1- and T2-weighted images, they appear as heterogeneous hyperintense nodal lesions relative to muscle tissue. These nodal lesions can invade surrounding structures. On computed tomography, it may contain coarse or branching-style calcifications [4]. However, there were no calcifications or signs of invasion in our case. The biggest dilemma of the unicentric subtype of Castleman's disease, as in this case, is the difficulty in ruling out possible metastatic lesions or other malignant processes in the presence of an existing space-occupying lesion. Owing to histopathological evaluation accurate diagnosis was achieved in this case. Thus, we believe that histopathological examination has an important role in indistinguishable cases.

## Conclusions

In conclusion, it should be kept in mind that the radiological manifestations of adrenal sex cord-stromal tumors can be confused with adrenal adenomas. Even when these extremely rare tumors are encountered, avoiding the "satisfaction of search" plays a crucial role in the identification of additional pathologies that can explain the etiology.
